# Approval of retinal gene therapies in the US and Europe based on visual acuity and microperimetry

**DOI:** 10.1080/17469899.2025.2487544

**Published:** 2025-04-03

**Authors:** Amandeep Singh Josan, Shabnam Raji, Robert Edward MacLaren

**Affiliations:** 1 Nuffield Laboratory of Ophthalmology, Department of Clinical Neurosciences, University of Oxford, Oxford, United Kingdom; 2 Oxford Eye Hospital, Oxford University Hospitals NHS Foundation Trust, Oxford, United Kingdom

**Keywords:** Clinical trials, inherited retinal disease, fundus-controlled perimetry, outcome measures, low luminance, regulatory, FDA, EMA

## Abstract

**Introduction:**

Gene therapy is an emerging technology for the treatment of inherited retinal diseases. Whilst the development of delivery vectors and genotyping is progressing at speed, outcome measures used for regulatory approval are slow to change and hinder progress clinical trial stage. Traditional measures of visual function such as best corrected visual acuity (BCVA) and low luminance visual acuity (LLVA) may only be useful across a very short window and in late stages of disease. They are unsuitable measures for early- to mid-stage disease where foveal function, and so letter reading, is primarily unaffected. In such cases, microperimetry is an accurate and repeatable measure of retinal function of the whole of the macular region.

**Areas covered:**

This article provides evidence-based guidance on criteria for microperimetry outcome measures, drawing on experience from long-term clinical trials in *RPGR-*related retinitis pigmentosa.

**Expert opinion:**

Microperimetry provides a sensitive and repeatable measure of retinal function, with mean sensitivity across the macula or central 16-point region offering a more reliable metric than single-point requirements recommended by the FDA. A 2.5dB gain in mean sensitivity is equivalent to a 13-letter increase in low-luminance ETDRS representing a clinically significant change and aligning closely with regulatory standards.

## 1 Introduction

Gene therapy research and development has grown rapidly in recent years and the number of clinical trials involving gene therapy products has correspondingly increased. Despite this, a limited number of gene therapies have been brought to the market successfully. Luxturna (voretigene neparvovec) was the first adeno-associated virus gene therapy in ophthalmology, approved by the U.S. Food and Drug Administration (FDA) in 2017 for *RPE65*-associated Leber’s congenital amaurosis (LCA) based on a novel outcome measure known as the Multi Luminance Mobility Test (MLMT) [1]. The following year the treatment was approved by the European Medicines Agency (EMA). New developments in outcome measures have remained stagnant and at present, the majority of clinical trials are powered by either best corrected visual acuity (BCVA) or microperimetry as the primary endpoint.

A 15-letter (3 line gain) gain in BCVA was originally adopted as an endpoint in the Diabetic Retinopathy study and Early Treatment of Diabetic Retinopathy study [2]. The FDA reason that a 15-letter gain is outside the normal range of measurement variability and represents a clinically significant change of doubling the angle of resolution [3,4]. The EMA have previously adopted a less stringent requirement of a 10-letter gain based on the more clinically applicable longitudinal link between visual acuity and health-related quality of life in patients with diabetic retinopathy [5]. Letter reading ability largely ignores the predominantly mid-peripheral presentation of many inherited retinal diseases (IRDs) and so BCVA will not measure clinically relevant characteristics in such cases where foveal function is preserved. Indeed, from our experiences of patients with retinitis pigmentosa and choroideremia, BCVA may be completely unaffected even in the presence of a dramatic reduction in quality of life. It is therefore clear that a change in letter reading ability is not always an adequate measure of disease progression or treatment efficacy. More recently, the FDA has accepted microperimetry as a clinical trial endpoint. A clinically significant gain is typically defined and agreed prior to commencement of the trial, but is generally defined as being 5 or more points within the central 16-point grid demonstrating a 7 dB gain in sensitivity. Notably, consensus on this outcome was reached based on studies of static automated perimetry in glaucoma, not microperimetry in IRDs [6].

In this article, we propose new evidence-based guidance on microperimetry as an outcome measure and answer three broad questions:

1) Is microperimetry mean sensitivity change a superior clinical trial outcome measure to single pointwise changes?
2) What level of microperimetry mean sensitivity can be considered ‘clinically significant’?
3) How should clinical trial successes and failures based on *any* functional outcome measure be reported?

Single pointwise criteria adopted by the FDA have little relevance in inherited retinal diseases. As with many IRDs, retinitis pigmentosa is primarily a disease of photoreceptors [7], whereas glaucoma is a disease of the retinal ganglion cells and their axons (nerve fibres). This distinction is relevant since small shifts in stimuli point position have little effect in glaucoma where retinal ganglion cells with wide receptive fields are being tested. By contrast, photoreceptor degenerations are vulnerable to a ‘cliff edge’ effect in which dramatic changes may be seen even with slight misalignment of stimulus point position. Treatment benefits in IRDs often manifest as contiguous gains across the central 16 points and beyond. This results in islands of functional recovery rather than few extremely focal point gains. The demarcated borders between functional island and scotoma pose challenges for interpreting treatment efficacy based on single-point analysis. Small errors in microperimetry point placements from one visit to the next can lead to dramatic changes in the assessment of retinal sensitivity and has previously been reported as the source of high pointwise test-retest variability [8]. As such, it is inappropriate to analyse single points and instead, global metrics should be favoured. The FDA criterion for a clinically meaningful improvement in visual function is at least 5 points gaining 7dB or more (total gain = 35dB minimum). Hence, a total gain of 35dB would be equivalent to a mean increase of 2.2dB spread across all central 16 points. A 2.2dB mean sensitivity gain across the central 16 is a more accurate and realistically achievable goal in the context of inherited retinal diseases, as more modest gains can potentially be captured over a greater number of central retinal points. Using the mean sensitivity value is beneficial as it mitigates any variability by averaging. Whilst this may seem undesirable in detecting focal treatment gains, the uncertain variability and potential for steep changes associated with scotoma borders using the current generations of microperimetry devices means that it is important to mitigate any variability by adopting global measures to avoid mis-interpreting pointwise changes resulting from noise as a treatment signal. This is distinctly different in static perimetry used in glaucoma studies where point changes are representative of far larger regions of the retina due to the large ganglion cell receptive fields and so pointwise variability has vastly different origins. The origin of the FDA criteria in studies of glaucoma and the lack of scientific support for a ‘5 point, 7dB change’ creates a false equivalence of repeatability in microperimetry with static perimetry. It is therefore incumbent on us to return to the question what represents a clinically significant and relevant change in microperimetry for IRDs?

## 2 An evidenced based criteria for microperimetry outcome measure

To fill this evidence gap, we determined a link between changes in low luminance visual acuity (LLVA) ETDRS letter reading (another measure of cone sensitivity) and microperimetry mean sensitivity of the central 16 points (MP16). This link aimed to offer guidance on defining a clinically significant change in microperimetry. Our analysis involved a retrospective, longitudinal examination of the first-in-human phase 1/2, dose-escalation masked clinical trial for X-linked RP caused by *RPGR* gene mutations (https://clinicaltrials.gov/: NCT03116113). Patients received subretinal delivery of an adeno-associated viral vector encoding codon-optimized human RPGR (AAV8-coRPGR), which expresses the fully glutamylated open reading frame (ORF) 15 of the RPGR protein –a region proven to be essential for cone function [9]. This analysis included 15 treated eyes from 15 patients, with up to 5 years of follow-up. Once unmasked to treatment we employed mixed model repeated measures (MMRM) to correlate changes in MP16 with LLVA, accounting for the longitudinal data structure and within-subject associations.

**Figure 1:**
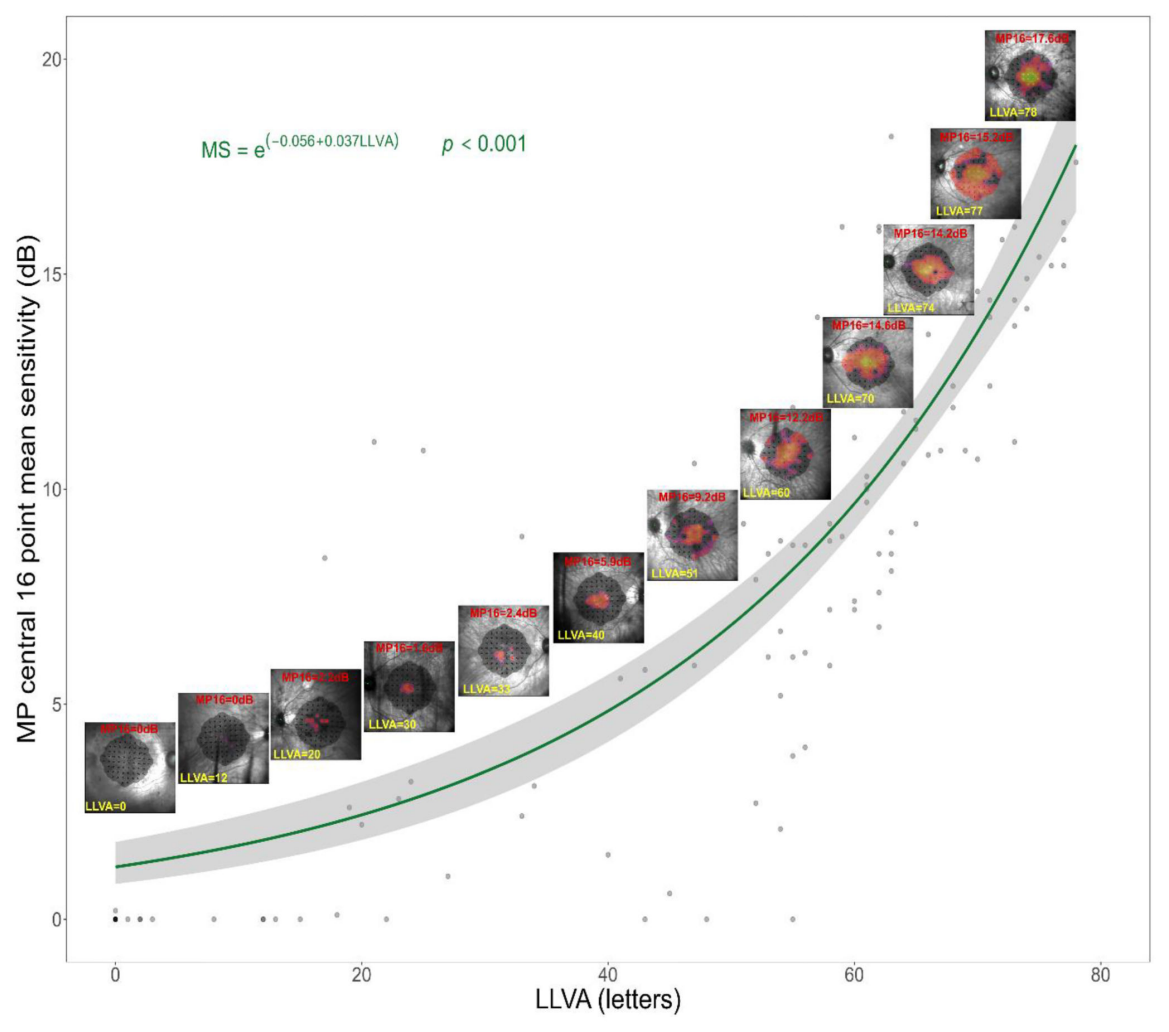
Exponential relationship between the central 16-point mean sensitivity (MP16) and LLVA (low luminance visual acuity) with exemplar microperimetry plots taken from a selection of treated eyes and timepoints across the long-term follow-up trial. Note that there is a floor effect in microperimetry (MP) in some patients as the maximal stimulus brightness is limited by the device’s capability. A hypothetical device with a wider dynamic range and brighter stimulus capability would likely eliminate the floor effect and result in an even tighter relationship between MP16 and LLVA.

For this cohort of *RPGR*-related RP patients who received treatment, the range of LLVA values were 0 to 78 ETDRS letters (Figure 1). As we would expect, MP16 does not follow a linear relationship with LLVA. The best fit is an exponential curve (p<0.001). This corroborates with previous work [10] showing that LLVA performance is predominantly dictated by the level of retinal function within the central most microperimetry points, that span an area of around 1-2 degrees radius. As such, at the lower end of this range even a very small gain in MP16 would be concentrated on the central 4 points resulting in an extremely large gain in LLVA. Conversely, at the upper end of this range, extremely large gains in MP16 tend to be concentrated on the para-foveal points and so have a minimal effect on the resulting LLVA score.

**Figure 2:**
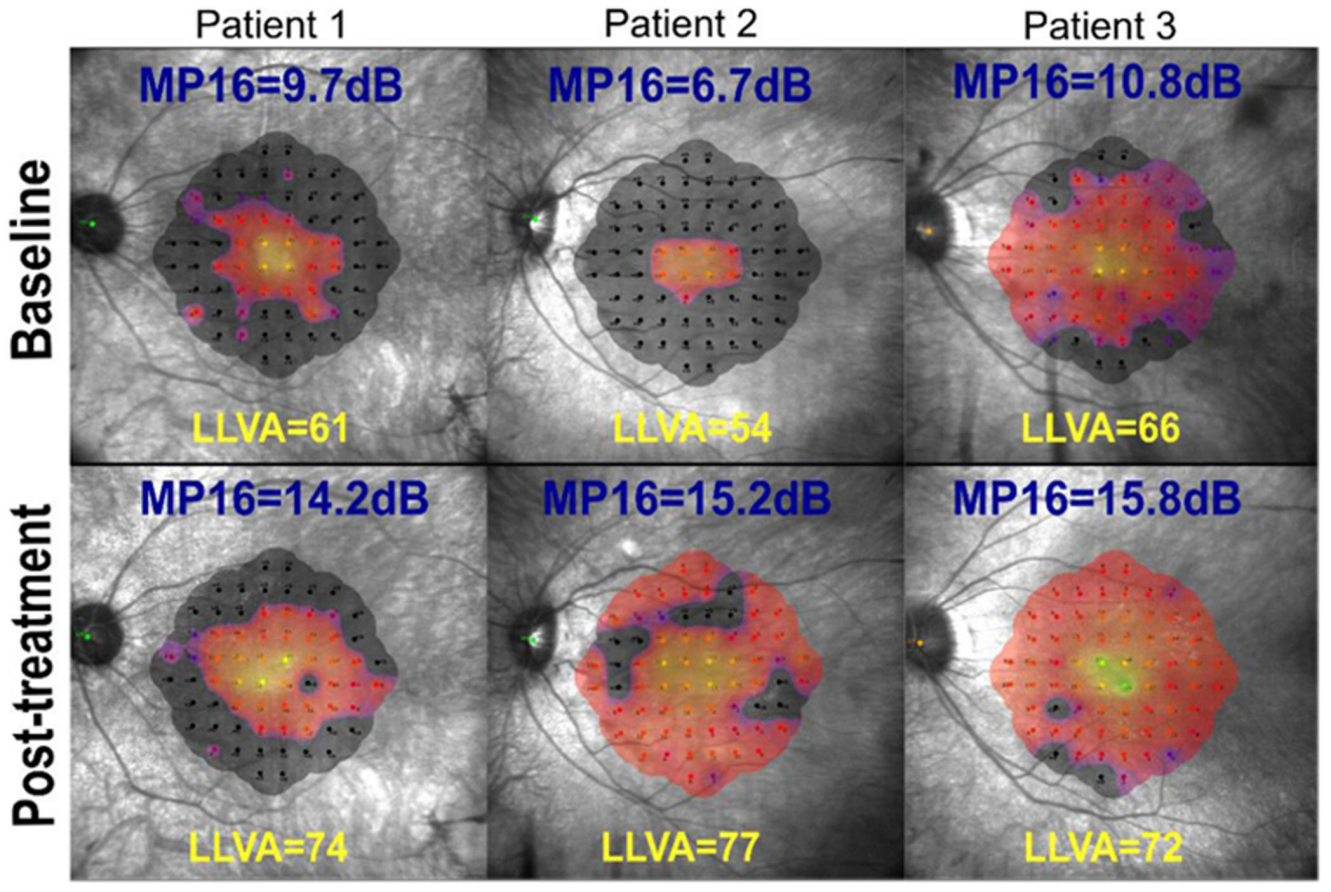
Microperimetry plots from three different patients at baseline versus 3-, 6- and 1-month post-treatment responses, respectively (bottom left to right), shown with corresponding central 16-point mean sensitivity and LLVA values. These patients highlight the shortcomings of using LLVA as an outcome measure as demonstratable increases in retinal sensitivity as a result of gene therapy do not always translate to commensurate gains in LLVA due to the ceiling effect observed in LLVA in patients with relatively preserved central retinal function Beyond this ceiling, LLVA measures no further useful information on gains in retinal function (right column – baseline LLVA at 66 is close to maximum and while only 6 letters were gained post-treatment, MP16 increased very significantly by 5dB). A solution to overcome the ceiling effect in patients with good baseline LLVA may be to exclude those with higher LLVA values, in a similar way to how BCVA was used in a recent choroideremia phase III trial [11]. Alternatively, microperimetry mean sensitivity in the central 16 points or beyond may be considered the preferred primary outcome measure in place of BCVA or LLVA.

This effect is illustrated with specific cases in Figure 2 demonstrating the sensitivity of LLVA to the central 1-2 degree region. It is important to note that this exponential relationship remains largely unchanged even when excluding patients exhibiting zero decibels of retinal sensitivity in the central 16 points (i.e. removing the MP16 datapoints that may be inaccurate due to late stages where the floor effect of the device has been reached).

To determine a clinically significant change in microperimetry mean sensitivity, we focus on patients with compromised but not extinguished low luminance visual acuity (LLVA). By correlating changes in LLVA with changes in microperimetry mean sensitivity in this subset, we derive a decibel value considered clinically significant based on the equivalent LLVA change. This value can then be applied universally, even for patients whose LLVA falls outside the range of measurable change. To identify meaningful changes in retinal function through low luminance visual acuity, a baseline LLVA score of 39 letters, representing the centre of the 0 to78 letters range, is considered. Using the equation of curve in Figure 1, a 2.2dB post-treatment gain (equivalent to the FDA-accepted 5 point, 7dB gain) in a patient with a baseline LLVA of 39 letters would result in a post-treatment LLVA of 51 letters, indicating a gain of 12 letters. Hence, this LLVA change aligns very nicely with the VA criteria from both the EMA and FDA, who consider clinically significant gains in visual acuity to be 10 or 15 letters respectively. Specifically, from a baseline value of 39 LLVA letters, a 10, 13 and 15 letter gain equates to a 1.8dB, 2.5dB and 3dB gain in MP16 respectively.

The authors wish to clarify the impact of a 2 to 3dB gain on LLVA based on different baseline LLVA scores. While a 2.5dB gain in a patient starting with a baseline LLVA of 39 letters results in a 13-letter gain, the effect varies for patients with baseline scores above or below 39 letters. This guidance aims to understand the implications of a 2.5 dB gain in peripheral regions by comparing when this level of gain is transferred to the very central 4 points. If a 2.5dB gain is deemed clinically significant in the central 4 points, it can also be considered meaningful in the broader central 16 points or beyond in regions where sensitivity gains do not translate to LLVA gains. This approach establishes an evidence-based mean sensitivity criteria applicable to all patients regardless of disease stage or initial LLVA ability, ensuring treatment success is not solely measured by LLVA which represents the central 4 points only. Whilst Figure 1 is established from patients with *RPGR*-related RP, we do not propose that the guidance obtained be restricted to those with this disease, but rather argue that *RPGR*-related RP serves as an ideal test case, due to its shallow sensitivity gradient, in order to investigate fundamental relationships between retinal sensitivity and LLVA in order to answer the question: what level of retinal sensitivity change is equivalent to a 2-3 line change deemed clinically significant beyond natural variability? Whilst there may be considerations in generalising in this way, such as in diseases where secondary rather than primary photoreceptor degeneration occurs, we argue that this is far preferable to the situation we have currently of generalising from static perimetry in glaucoma studies. Further work investigating a variety of ocular diseases would be beneficial to establish whether the this relationship between LLVA and retinal sensitivity is valid across a broad disease range or if adjustments are warranted.

Whilst microperimetry offers a comprehensive assessment of retinal function beyond the scope of low luminance visual acuity (LLVA), we must question whether patients truly benefit from changes in LLVA or retinal sensitivity? It can be assumed that there are significant advantages to gains in both retinal sensitivity in foveal regions (important for LLVA) and those in peripheral regions (which do not manifest as increases in LLVA). This assumption could be definitively answered with alternative measures of therapeutic success such as patient reported outcome measures (PROMs).

Improvements in quality of life (QOL) can be considered the ultimate goal of any therapy but accurately assessing this is a notoriously difficult task as data collected is often fraught with noise. PROMs, such as the VFQ-25 among others [12,13], can serve as an alternative measure of therapeutic success by aligning changes in visual function with quality of life. However, PROMs are often non-specific and challenging to tailor to specific diseases or severity levels. Mobility assessments such as the MLMT provide an additional measure of functional vision in varying light levels and primarily assess rod photoreceptors. However, significant barriers, such as investment in space, technology and training, limit its widespread use. In addition, tests of rod photoreceptor function can be highly useful in diseases such as retinal dystrophies caused by mutations in RPE65 where there may remain many surviving rods that are unable to dark-adapt without the RPE65 enzyme. Restoration of RPE65 function here would likely lead to a significant gain in rod function and thereby, scotopic vision. With RPGR and other retinal degenerations in which loss of rod function is predominantly caused by loss of actual rod photoreceptor cells, the capacity for significant improvements in rod (scotopic) vision after replacement of the missing gene may be limited.

Regardless of these drawbacks, any new test that is able to capture and quantify difficulties in daily life is a welcome addition. Similar to PROMs, however, data obtained from mobility assessments are prone to noise from natural variability and confounding effects, such as learning and motivation in patients who know that they have been dosed. In RPGR and other retinal degenerations involving photoreceptor loss where significant rod recovery is unlikely, small to moderate improvements in cone function are sought and these may not be discernible from measurement variation produced by the motivational or learning effects in patients using these assessments. As a result, a larger number of patients would be needed in clinical trials to eke out any difference between doses in order to provide a reliable evaluation of treatment efficacy.

**Figure 3:**
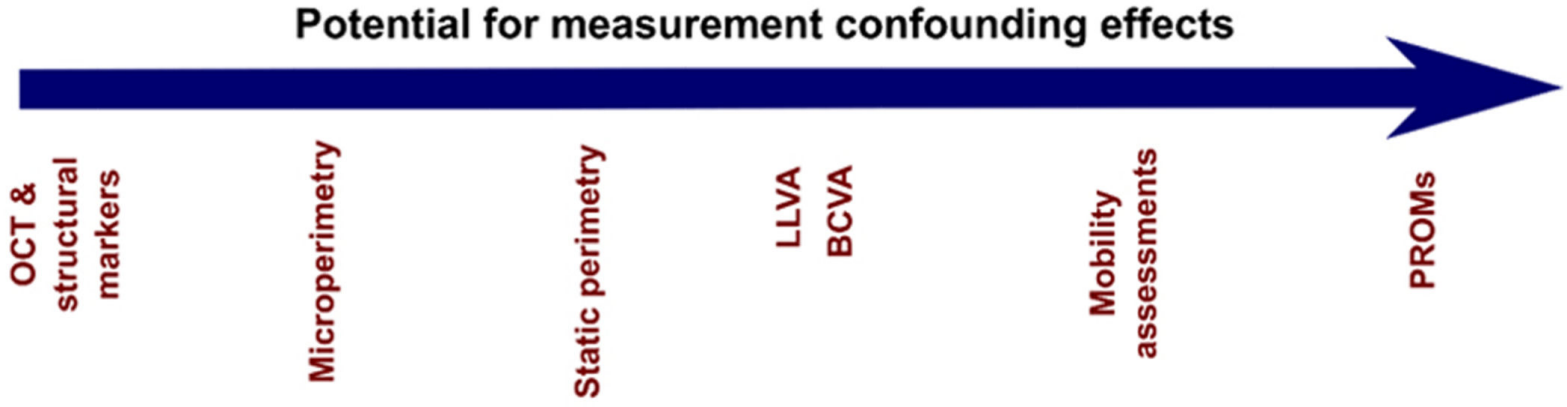
Ordered heuristically from least to most potential for measurement errors due to confounding effects, such as learning, motivation and confidence, with the least potential on the left and the most on the right. These confounding effects pose a higher risk of false positive and false negative interpretations, potentially masking true therapeutic effects or falsely attributing measurement changes to the investigational medicinal product. Outcome measures can be considered more relevant and patient-centred towards the right as they more overtly link to an improved quality of life —the ultimate treatment goal.

Any assessment of visual function will suffer from some degree of natural variability, whether it be from tiredness, learning effects or some other unknown factor that is independent of a true change in visual function. This is perhaps most relevant for mobility tests where, despite best efforts, confounding factors are far more difficult to control than in microperimetry or ETDRS letter reading because only one variable is being tested (navigation accuracy). To ensure meaningful assessments, it is critical to consider test-retest variability at baseline for the specific cohort under investigation. Any reported therapeutic gains should be evaluated in the context of expected natural variability, similar to the standards applied in glaucoma research. Just as a new intra-ocular pressure device would require a measure of test-retest variability, any approved treatment for visual function should demonstrate efficacy beyond natural variability [14,15]. Undeniably, for tests such as the MLMT that are vulnerable to learning, motivation and confidence, there will be a significant placebo effect for patients who believe they have just received a treatment for their incurable disease. Equally, it is impossible to control for the psychological effects in a mobility test in which the control group participants are fully aware that they did not receive a treatment. For tests that are dependent on higher cognitive function, a true sham effect needs to be factored in, as measurements from these tests may be dependent on psychological factors as much as vision. Outcome measures based on highly subjective tests such as these, whilst appealing, demand a greater proof of concept in terms of potential confounding effects than more objective measures (figure 3).

## 3 Conclusion

Based on our experience in pivotal clinical trials for inherited retinal degenerations, microperimetry stands out as a preferred measure of visual function, surpassing BCVA or LLVA. This view is supported by the increasing use of microperimetry as a primary outcome measure in various clinical trials [16]. Advantages of the test include excellent reproducibility, short testing times, low test-retest variability, and automatic control for potential confounding effects through blind spot testing, fixation monitoring, and reliability metrics. The test output is easily interpretable and highly sensitive to minor changes in retinal function. The microperimetry test mean assesses responses to 68 different points which reduces the effect of an error on one test point. While LLVA is more patient-friendly for self-monitoring, it does not demonstrate sensitivity in detecting small treatment effects. Mobility assessments can introduce more noise in gathered data due to increased cognitive requirements which introduce psychological variables. The shortcomings of LLVA and mobility courses outlined above are adequately compensated for with microperimetry.

To expedite gene therapy delivery to market, outcome measures demonstrating treatment efficacy and predicting clinical benefit are crucial. We assert that microperimetry is optimal for assessing true therapeutic gains and offer updated guidance on what level of retinal sensitivity gain should be deemed clinically significant.

## 2 Expert Opinion

Gene therapy holds transformative potential for inherited retinal diseases, but its advancement is hindered by reliance on outdated or inappropriate outcome measures like best-corrected visual acuity (BCVA). Microperimetry offers a more accurate, sensitive, and clinically relevant assessment of retinal function, especially for IRDs where mid-peripheral degeneration predominates and foveal vision remains preserved. However, while stimuli placements in microperimetry are vastly more accurate and reproducible than those in traditional static perimetry (due to the presence of live fundus-tracking), the single points are still prone to a degree of placement errors. In the presence of steep gradients of sensitivity between functioning and scotomatous retinal regions these placement errors can lead to significant variability in single pointwise threshold measurements. Unlike single-point criteria currently endorsed by the FDA, mean sensitivity metrics across the central 16-point grid provides a robust and reproducible measure of treatment efficacy, mitigating noise caused by pointwise variability. Our findings suggest that a mean sensitivity gain of 2.5 dB, equivalent to a 13-letter gain in low luminance visual acuity (LLVA), is a reasonable threshold for defining clinically meaningful outcomes. This updated guidance aligns with both EMA and FDA criteria for visual acuity gains while accommodating the specific challenges of IRDs. These changes to regulatory guidance based on established microperimetry have the benefit of being extremely simple to adopt and could significantly benefit research into new therapies. The implementation of clear and evidenced-based criteria for outcome measures in IRDs would assist in bringing novel therapies to market with associated significant benefit to patients. In addition, any approval based on a reliable and sensitive outcome measure such as microperimetry mean sensitivity would inevitably spur further potential treatments and research for currently untreatable ocular diseases. The situation of ambiguity and suboptimal outcome measures currently approved by regulatory agencies introduces an unwarranted risk of failure based solely on an unsuitable choice of clinical trial endpoint.

Key improvements in this field should include adopting global metrics such as mean sensitivity for clinical trials, refining microperimetry devices to expand their dynamic range and eliminate floor effects, and enhancing accessibility to such technologies. Additionally, integrating patient-reported outcome measures (PROMs) tailored to IRDs could bridge the gap between functional gains and quality-of-life improvements, though these tools remain prone to variability and require disease-specific refinement. While PROMs and mobility assessments may complement functional testing, their susceptibility to cognitive and psychological biases underscores the necessity of measures like microperimetry as primary trial endpoints. Further research into optimizing PROMs for use as an outcome measure by establishing robust structure-function-QOL associations would greatly aid in bringing novel therapies to market in future. However, in studies that do not involve a sham procedure in control groups, the potential for significant confounding factors such as patient knowledge of treatment and resulting biases may prove impossible to overcome. More specifically, in potential therapies for RPGR-related retinitis pigmentosa and other similar retinal degenerations involving photoreceptor loss, where significant photoreceptor recovery is likely to primarily involve cones rather than rods, mobility testing may not provide a sensitive test of treatment efficacy, In the next five years, we anticipate a shift in IRD clinical trials toward harmonizing outcome measures across regulatory bodies, with microperimetry mean sensitivity emerging as a standard. Clinical trial designs may increasingly emphasize global retinal function rather than focal gains, allowing for more inclusive assessments of therapeutic efficacy.

## Article highlights

- Microperimetry stands out as a sensitive, repeatable, and clear measure of retinal function.
- Single point requirements such as the 5-point 7dB gain required by the FDA are subject to variability in IRDs and should be abandoned in favour of a mean sensitivity of the entire macula region, or within the microperimetry central 16-point region
- A 10,13 and 15 LLVA letter gain equates to 1.8dB, 2.5dB and 3dB gain in microperimetry mean sensitivity within the central 16-point region respectively. Hence, a 2.5dB gain, equating to a 13-letter increase in low luminance ETDRS, would be a reasonable definition for a ‘clinically significant’ change being more stringent than the FDA’s current single-point requirement and mid-way between EMA’s 10 letter, and FDA’s 15 letter ETDRS requirements.
- In regions ***not*** centred on the fovea, a 2.5dB mean sensitivity gain should still be viewed as clinically significant, irrespective of changes in letter reading ability. Because these treatment gains can be deemed to be equivalent to a 13-letter gain if centred on the fovea.
- All potential outcome measures, especially mobility assessments, should include and report patient specific test-retest variability for treatment benefits to be validated as true gains beyond natural variability and should be interpreted with caution where there are small gains in patients who have not been subjected to a sham procedure and are aware that they have received a treatment.
